# High Resolution Trichromatic Road Surface Scanning with a Line Scan Camera and Light Emitting Diode Lighting for Road-Kill Detection

**DOI:** 10.3390/s16040558

**Published:** 2016-04-19

**Authors:** Gil Lopes, A. Fernando Ribeiro, Neftalí Sillero, Luís Gonçalves-Seco, Cristiano Silva, Marc Franch, Paulo Trigueiros

**Affiliations:** 1 School of Engineering of the University of Minho, Guimarães 4800-058, Portugal; gil@dei.uminho.pt (G.L.); cristiano.c.s@gmail.com (C.S.); apoarmatu@gmail.com (M.F.); ptrigueiros@gmail.com (P.T.); 2 Geo-Space Sciences Research Centre, Observatório Astronómico Prof. Manuel de Barros, University of Porto, Vila Nova de Gaia 4430-146, Portugal; neftali.sillero@gmail.com; 3 Department of Communication Sciences and Information Technologies, University Institute of Maia, Avioso São Pedro 4475-690, Portugal; lgseco@gmail.com

**Keywords:** road scanning, computer vision, pavement inspection, line scan camera

## Abstract

This paper presents a road surface scanning system that operates with a trichromatic line scan camera with light emitting diode (LED) lighting achieving road surface resolution under a millimeter. It was part of a project named Roadkills—Intelligent systems for surveying mortality of amphibians in Portuguese roads, sponsored by the Portuguese Science and Technology Foundation. A trailer was developed in order to accommodate the complete system with standalone power generation, computer image capture and recording, controlled lighting to operate day or night without disturbance, incremental encoder with 5000 pulses per revolution attached to one of the trailer wheels, under a meter Global Positioning System (GPS) localization, easy to utilize with any vehicle with a trailer towing system and focused on a complete low cost solution. The paper describes the system architecture of the developed prototype, its calibration procedure, the performed experimentation and some obtained results, along with a discussion and comparison with existing systems. Sustained operating trailer speeds of up to 30 km/h are achievable without loss of quality at 4096 pixels’ image width (1 m width of road surface) with 250 µm/pixel resolution. Higher scanning speeds can be achieved by lowering the image resolution (120 km/h with 1 mm/pixel). Computer vision algorithms are under development to operate on the captured images in order to automatically detect road-kills of amphibians.

## 1. Introduction

Roads have multiple effects on wildlife, from animal mortality, habitat and population fragmentation, to modification of animal reproductive behavior. Amphibians in particular, due to their activity patterns, population structure, and preferred habitats, are strongly affected by traffic intensity and road density [1,2]. On the other hand, road-kills studies and conservation measures have been extensively applied on highways [3], although amphibians die massively on country roads [2], where conservation measures are typically not applied.

Portugal has no national program to control deaths on the road, a common practice in other European countries (e.g., United Kingdom and The Netherlands). This is required to identify road fatality points in order to correctly implement conservation measures [4]. However, the monitoring of these deaths is time consuming and expensive, especially depending on volunteers. Therefore, inexpensive, easy to implement, and automatic methods for the detection of road deaths in larger areas (wide monitoring) and over time (continuous monitoring) are required.

**Figure 1:**
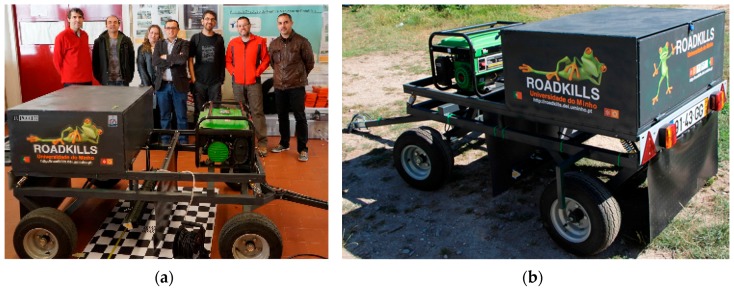
Developed road scanning trailer: (**a**) Prototype and project team; (**b**) Prototype outdoors.

Under the Roadkills project—“Intelligent systems for survey of mortality in amphibians Portuguese roads”, a joint venture of the University of Minho, University of Porto and University Institute of Maia, a mobile system was developed that is capable of acquiring images of Portuguese road surfaces in a continuous manner and over a pre-determined path defined by biologists. After several discussions on what type of vehicle would be suitable for this purpose, it was opted for the development of a mobile two-axle trailer as shown in Figure 1.

The type of the chosen solution was based on different criteria factors such as: (a) mobility; (b) versatility in being easy to attach to any vehicle; (c) motion stability needed for the image capturing system and thus the dual axle system; (d) standalone powering over several hours of utilization; (e) accommodation of all the equipment with protection to rain and dust; (f) capability to scan the road surface with high resolution of a complete track (~25 km) at an acceptable speed. Different approaches for car adaptation were taken into consideration but they were declined, due to different constraints (mechanical, electrical, legality, *etc.*). Also, the high power light emitting diode (LED) lighting utilized in the system would damage the car alternator due to its energy consumption. Thus, an external power system would be necessary for long runs and typically they are based on combustion engines (e.g., electrical power generators using fossil fuels). The last reason for this choice was the lack of independence created by a dedicated adapted car. On any engine failure, maintenance or any other possible reason, the whole system would come to a halt. Therefore, an independent and energy- autonomous trailer was the preferred choice. This paper describes the technical details of the developed scanning system, showing some results of the different tests conducted and a discussion, including a comparison with other existing systems.

### Related Work

Camera-based systems for road inspection are not a novel idea in itself [5]. There are specialized vehicles that use cameras or lasers to acquire accurate visual data from the road surface. Nowadays, there are some solutions based on vehicles which are adapted for road crack detection, with similar characteristics between each solution. These systems typically use area or line scanning cameras with an image resolution that allows the detection of cracks wider than 1 mm. The differences among them are mainly the width of the scanned area, ranging from 2 to 4 m [6,7]. Some known available systems are the Road Crack Detection from the Australian Commonwealth Scientific and Industrial Research Organization (CSIRO) [8], the Fugro Roadware’s Automatic Road Analyzer (ARAN) [9] and WayLink’s Digital Highway Data Vehicle (DHDV) [10]. In Europe, the PAVUE system [6] is operated in The Netherlands and Finland. This system can be equipped with either multiple video cameras or line scan cameras for the acquisition of continuous images of the road surface. Highways Agency Road Research Information System (HARRIS) [6] is another system developed as a result of 10 years’ research program carried out by the Transport Research Laboratory [6] of the United Kingdom. Laser Road Imaging System (LRIS) [11] was the name given to the system developed by a Canadian company named INO (Québec, QC, Canada) for longitudinal and lateral road cracking detection based on laser imaging systems. It uses both high speed/high resolution line scan cameras in conjunction with high power laser line projectors that are aligned in a symmetrically crossed optical configuration. The two-line scan cameras and lasers are configured to image 4 m transverse road sections with 1 mm resolution (4000 pixels) or 0.5 mm (8000 pixels’ option) at speeds that can reach 100 km/h. Using high power laser line projectors and special optics, the LRIS system can operate in full daylight because it minimizes the effects caused by variations in outside lighting conditions and shadows cast by roadside objects, viaducts and the inspection vehicle itself. For the domain of inspection of transportation infrastructures, the exclusive client for the LRIS is Pavemetrics Systems Inc. (Québec, QC, Canada) [11]. ICC (Lakeland, FL, USA) is the name of another company that has developed a technique similar to the LRIS system [12].

Laser technology is used for road crack detection and pavement assessment. Its application can be divided in two categories: (a) as an illumination system for single channel (shades of gray) line scan cameras [6,13]; (b) as a profile scanning system providing depth information of the road surface [14,15]. In the case of the Roadkills project, the amphibian detection algorithms require high resolution color images for the process of image recognition. Some features of the amphibians can be detected by examining the color of the animal skin and also by the detection of the shape of legs, feet and toes (hind limbs and its parts with sizes below 1 mm) [16]. Thus, single channel images are not sufficient. On the road, the animal is flattened by car crushing and hence, the profile measurements using laser scanning may not retrieve relevant information for animal detection by the computer algorithms. Therefore, laser technology was not considered on this project.

**Figure 2:**
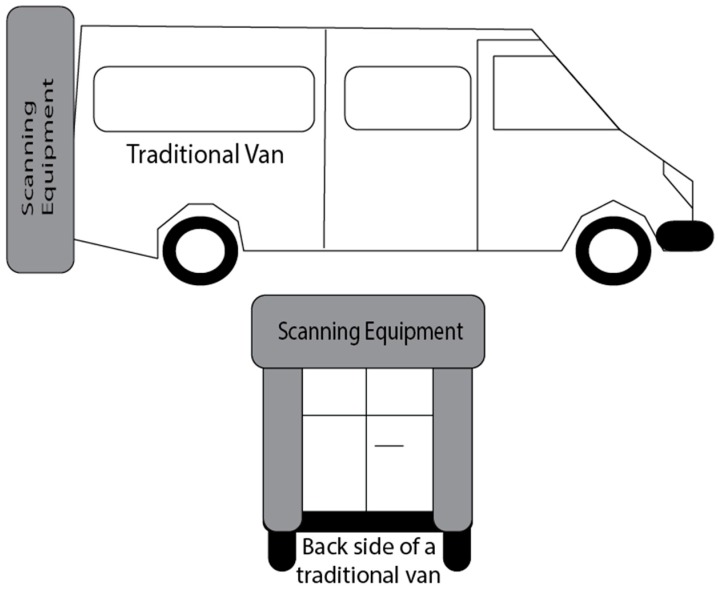
Example of the adaptation found in the existing road scanning systems.

Figure 2 shows an example of how typically vehicles are adapted on the described systems. They are essentially based in adapted commercial vans. The cost of the vehicle itself plus the adaptation can go up to around several hundred thousand euros [5]. The process is considered accurate, but too expensive for many road maintenance agencies and therefore it is performed only once every two years or so [5]. This budgetary level is too high for the purposes of this project and therefore, it was necessary to search for valid solutions that could be carried out at much lower cost.

## 2. Materials and Methods

As explained before, the developed prototype was based on a dual axle trailer that can be easily towed by any towing system attached to a traction vehicle (car, van, jeep, quad bike, truck, *etc.*). The electrical connection to the traction vehicle is used only for the vehicle’s rear light connections (tail lights, indicators and number plate). Next the details of the developed prototype are described.

### 2.1. System Architecture

**Figure 3:**
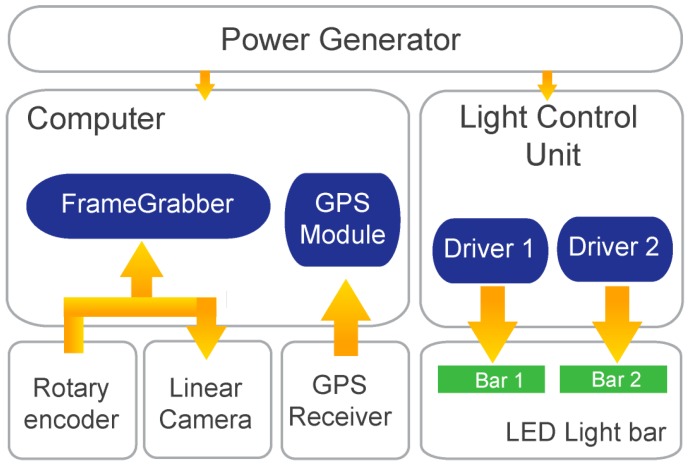
System architecture of the developed solution.

A wide variety of devices are set up in the trailer in order to acquire the required information. An imaging system is used for acquiring road surface images. A computer is used for controlling the device, storing data and processing it offline for obtaining information about the road state. A GPS is used to obtain the coordinates for all acquired frames. Although a line scan camera is used, acquisition from the camera is performed in frames or groups of lines. Finally, all these devices are synchronized using an encoder attached to the left rear wheel of the trailer. The block diagram of Figure 3 shows the system architecture of the developed solution.

On top of the diagram of Figure 3 is the electrical power generator running on petrol and supplying 2.2 kW of alternating current (AC) 230 V at 50 Hz, allowing the system its independence from any external power supply. As long as petrol is provided to the generator, the system runs uninterruptedly. A 12 V battery (190 Ah) attached to a 2 kW pure sine wave inverter was also tested, instead of the petrol generator, providing enough power for a 5 km run at an average trailer capturing speed of 30 km/h. Since the whole system is paced by motion due to the use of an encoder, when the trailer is stopped the image acquisition is also stopped. This allows the user to refill the tank of the power generator, allowing some more hours of image acquisition. From the power generator two leads with switches enable both systems (computer and LED lights) to be turned on separately. If the trailer is stopped and the trailer’s computer is being accessed, the LED lights can be switched off.

The incremental rotary encoder provides the needed synchronization of the different devices of the system, namely the camera line acquisition and hence the GPS localization coordinates acquisition. It is based on a Telemecanique XCC1510PS50X (Schneider Electric, Andover, MA, USA) with 5000 pulses per revolution [17] connected directly to the wheel hub with no divider or multiplier. On each wheel turn 5000 pulses are generated and hence the same amount of lines can be captured. Since the encoder is connected to the frame grabber board it is thus controlled by it. Whenever the user sends the record command via a software interface developed for this purpose, the frame grabber uses the encoder pulses to get camera lines and to download a complete frame or set of lines. At the same time, for each acquired frame (group of lines) a GPS coordinate is obtained and both data are saved to disk.

In order to acquire proper images of the road surface, the camera should provide high enough data acquisition and transfer speeds so that the short exposure times is enough to produce significant output values, with an external and controlled light source. An analysis performed on different cameras, showed that a good camera option would be a line scan camera, commonly used in industry and in controlled environments. This type of camera can operate at very high data rates when compared to area scan cameras or frame cameras. In a continuous mode, only a single line is scanned at a time perpendicular to the road path whereas with an area scan camera (frame camera) a whole frame is capture at a time and one must ensure that at different vehicle speeds, there is always some overlap between images. A perfect match without overlapping with line scan cameras is difficult to attain due to non-controllable hardware synchronization delays. Hence the line scan process becomes more effective and faster for this type of application. Also, the typical spherical lens distortion (barrel distortion) in a frame camera needs correction on every frame for a proper image stitching. This is not necessary on line scan cameras since the continuous line by line scanning along the road path does not produce such a feature. Any image correction is only longitudinal and constant on each captured line.

Suitable lighting is required to obtain high quality images and provides a more controllable environment outdoors, during a sunny day or at night. The most usual lighting sources are the halogen lamp, light emitting diodes (LEDs) and lasers. Halogen lamps require large amounts of energy for the same light intensity as other sources of light, since it is a less efficient process. LEDs have better power efficiency than halogen lamps, but worse efficiency than lasers. However, the choice fell on the LED illumination option as compared with laser light because white laser light is not commercially available. White laser technology was recently developed [18] and it will take some time until it is available commercially. White light is essential for color image capture in order to avoid chromatic distortion. To operate with color image, acquisition the LED lighting was in fact chosen as becoming the best compromise between all the described factors. Although more efficient than the halogen lamps, the LED unit used has still a power consumption of 640 W.

The application of a line scan Red-Green-Blue (RGB) camera (also known as trichromatic camera) with the LED lighting system, outdoors and in a moving vehicle, is innovative given that these systems are commonly used in industrial controlled environments. Many different problems had to be foreseen and accounted for such as energy, vehicle stability and constant distance to the ground to maintain proper camera focus. Computer processing, system usability and environment protection are also issues that were accounted for. The system has to scan several kilometers of countryside bumpy road surfaces, having to produce an accurate and georeferenced high-resolution sequence of images for post processing, in order to detect and localize amphibian casualties. Only 1 m of road width is scanned with the developed system. To allow a wider road scanning, different solutions can be implemented as further explained below.

### 2.2. Equipment Description

The developed solution is modular and contains two major blocks, as described earlier. The scanning block is the core part and it has a model Piranha 4 line scan camera from Teledyne Dalsa (Waterloo, ON, Canada), with a single line resolution of 4096 pixels (24 bits’ color) at a clock frequency of 35 kHz. It is based on a Complementary Metal-Oxide-Semiconductor (CMOS) sensor [19]. The camera uses a 35 mm Nikon lens, positioned at a distance of 0.85 m from the road surface. At this distance, a full scanned camera line has 1 m width of the road surface. Since the camera and lens is protected from the environment (dust and rain) by the trailer box, the distance to the ground is important to avoid the necessity of having a taller box. This can create instability when circulating on the road, due to lateral and frontal winds. The camera is connected to a Teledyne Dalsa model Xcelera-CL PX4 Full frame grabber. This frame grabber is installed into an industrial computer with a ASUS Intel Z87 motherboard, Intel Core i7-4770 processor, 16 GB Random Access Memory. Data storage is performed by three Solid State Disks (SSD) of 512 GB each with an extra SSD of 128 GB for operating system and developed applications. Solid State Disks were chosen to avoid mechanical disruption from the trailer vibrations, caused by the trailer movement on countryside roads. Since the Dalsa software development kit is only available for the Microsoft Windows platform, version 7 of this operating system was used with Microsoft Visual Basic as the development environment and language.

One incremental rotary encoder is attached to the left rear wheel. It is a model Telemecanique XCC1510PS50X from Schneider-Electric, with 5000 pulses per revolution (ppr) and IP65 protection [17]. The IP code stands for the International Protection Marking, from the International Electrotechnical Commission (IEC) with the standard 60529. The GPS device used was the Ublox 7 Evaluation kit for precise point positioning, delivering less than 1 m error. The system uses the National Marine Electronics Association (NMEA) standard coordinates [20] for frame capturing localization. For the light block the chosen equipment is the Corona II—LED Line Scan Illumination from Polytec [21]. With 1 m in length, this light unit provides linear polarized cool white light (5000 K) with IP54 protection. The unit needs a dual drive controller to provide its 640 W of power at 24 V. The light is converged to a straight line demanding some mechanism for focal point adjustment.

### 2.3. Calibration Procedure

**Figure 4:**
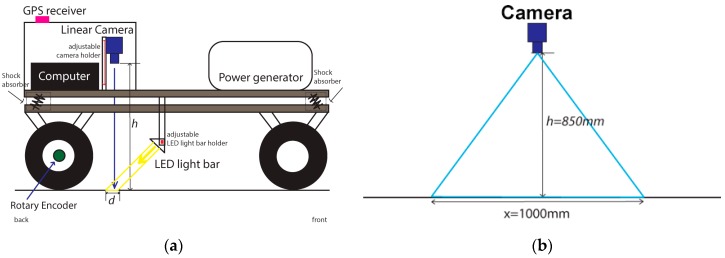
Diagrams of the developed scanner: (**a**) Complete device set with the blocks that makes the system; (**b**) Camera height and captured line width.

Due to the application and integration of different subsystems, individual calibration of each one was necessary in order to make the whole system fully operational. The trailer was designed and built with a dual axle, to allow a controlled and consistent distance from the camera position and its perpendicular point to the road surface. It is a very important requirement because in this way, there is no horizontal rotational movement (tilting) that occurs on one axle trailers. When attached to different height towing vehicles or during travelling on irregular bumpy roads, this tilting effect is constantly felt. Perpendicularity is then strongly enforced between the camera and the road surface. Figure 4a shows how the camera and the LED light bar unit were positioned, along with the other equipment described earlier. Both devices (camera and light) have adjustable fixtures allowing some fine tuning in different axes. For the camera, the main adjustment is its distance to the road surface whereas with the LED light, distance to the road and tilting are essential for a good alignment and positioning as shown in Figure 4a. This means that the line scanned by the camera should illuminated evenly throughout the 4096 pixels, to avoid changes in brightness and possible occlusions. Figure 4b shows the distances utilized for the camera positioning.

**Figure 5:**
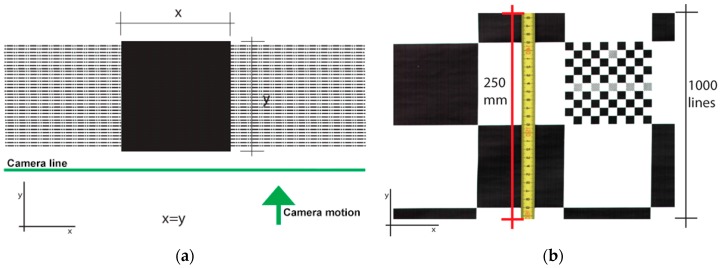
Scanning procedure using the line scan camera: (**a**) Checkerboard black square and respective scanning lines; (**b**) Captured lines and respective length in millimeters.

Another important subsystem calibration, is what defines the ratio between width and height of a scanned square on the road. In other words, as the trailer moves, the scanned lines must guarantee proportionality between the width (scanned line) and the length travelled (height of a scanned image) to avoid ratio distortion. This involves finding the relation between the incremental encoder pulses generated as the trailer moves and the trailer wheel perimeter. Thus, a calibration procedure was adopted that attempts to minimize the maximum possible errors in the proportionality of a scanned image. For such task, a checkerboard printed paper was laid down on the floor and scanned over, ensuring that it was aligned with the camera acquisition line. The scanned checkerboard was then analyzed and a single black square was isolated for the measurements, as shown in Figure 5a. As it can be seen, each scanned line defines one-pixel height of an image. Counting the obtained *x* pixels of the square (fixed by the camera line pixel distance), the same amount of *y* pixels should be attained (depending on the incremental encoder pulses). Dividing both values, a ratio is obtained and passed to the software setup, in order to define the pulses to use from the incremental encoder in the frame grabber. After the ratio is set, the hardware automatically accepts only the valid pulses from the encoder rejecting all the others.

The construction of an image or a frame is obtained through the acquisition of several consecutive lines. This is defined as a frame buffer and can be set with different lengths in the camera firmware setup. These non-overlapping continuous frames were tested with different lengths in order to understand recording speed differences and file sizes (e.g., 4096 × 1000 to 4096 × 4096 pixels). Although the scanning is continuous, each acquired set of lines or frames are stored locally on the computer disk storage as separate image files. If a file allocation table corruption occurs or any other similar problem, the chances of losing all the scanned track is minimized by losing only just the corrupted parts. A single file for the whole track is more difficult to handle. It cannot be easily transferred or opened in any digital image preview program due to its file size. A 24 bits RGB frame of 40,964 × 1000 pixels produces a raw file size of around 12 MB. The 4096 pixels defined as one scanned line is equivalent to 1000 mm image width (Figure 4b). With the calibrated length ratio, 1000 scanned lines is equivalent to 250 mm as shown in Figure 5b. For an average scanning run of 25 km, the system will store 100,000 files needing 1.48 TB of available disk space. Data storage throughput was another constraint found. At a travelling speed of 20 km/h, the system needs to store 346.6 MB/s of raw image file. By testing a lossless compression scheme with high quality output such as the Joint Photographic Experts Group (JPEG) format, the necessary throughput was reduced by 50%–60%. This alleviated hard disk storage throughput and no visible loss of image quality was observed.

Along with the recording of each frame, a text file is built where each line added corresponds to the sequential image file name of the recorded frame, followed by the NMEA sentence obtained from the GPS device with geographical coordinates and GPS precise time. This provides the information of where each frame was acquired and the respective date and time, for post-processing of the acquired image files.

## 3. Results

A series of road scans were conducted in order to test the developed system and software. Several routes were chosen and the system was experimented at different traveling speeds from 0 km/h to 40 km/h. In total, hundreds of kilometers were covered during all the performed tests and the data was carefully analyzed after each trip.

### 3.1. Experimental Setup

**Figure 6:**
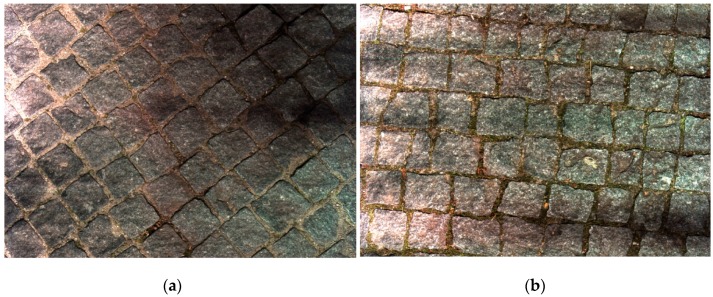
Scanning results in different situations: (**a**) Sunny daylight time; (**b**) At night.

The first set of tests was conducted for stress and robustness analyses, alongside the system failure finding. It has passed all performed day and night tests, on heavily bumpy cobbled roads. Images were acquired during these tests and it showed that at any travel speed, images were acquired with good quality (no tremor). No difference was found between the images acquired during the day and at night (Figure 6). It was then concluded that the lighting unit was performing well enough and it was sufficient for the purpose. From the other performed tests, the prototype revealed to be sufficiently robust to travel at the desired speeds on severely damaged roads. It also demonstrated accuracy on the obtained values, both from the GPS and the acquired images.

The second set of tests was made at lower speeds (below 40 km/h) and on asphalt roads. They were performed at different speeds of around 20 km/h and above, for continuous image acquisition. This would provide some feedback on the possible bottlenecks calculated around this travel speed, related to data throughput necessary to record the information. It would also provide information about any lines that could be lost in between each captured frame. Any lines lost would produce a gap between frames and jeopardize the computer vision detection algorithms. Due to the small size of the recognizable features of a crushed amphibian (e.g., hind limbs), one lost line may be sufficient to cause a misidentification by the software. The initial tests showed some gaps at low speeds and the gap size was consistent with the travel speed. After changes made to the software and some laboratory tests the gaps were removed. Only road tests at higher speeds could confirm the existence or not of gaps.

**Figure 7:**
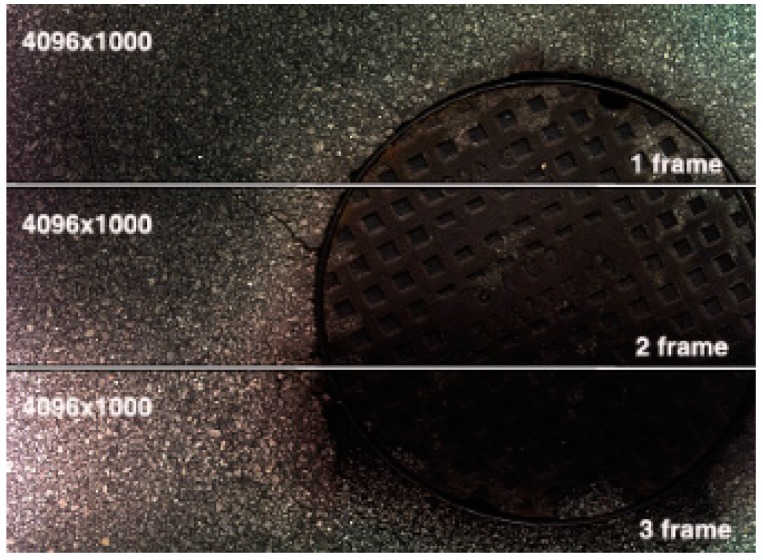
Three consecutive frames with a resolution of 4096 × 1000 pixels on each frame obtained at a travel speed of 20 km/h.

Several tests were carried out with different configuration parameters of the camera. Frame buffer (number of lines per frame) was changed between 1000 and 4096 lines to test for possible differences in the output data. The images obtained from the tests revealed good results with the tested values. At the same time, no line gaps were obtained at any tested travel speed. Figure 7 shows an example of three consecutive acquired frames with 1000 lines per frame at 20 km/h travel speed. The three frames of the figure were deliberately slightly detached to show their boundaries. When put together there is a perfect match and no gap or lost line is found.

After obtaining the images from the tests they were analyzed for quality inspection and frame sequence check. A playback software was developed to help on the analysis of image sequences by joining three or more frames on a single framed window. This allows a smooth scroll of the joined frames and a quicker inspection of any disturbance on road scanned images. In fact it allowed the detection of the predicted bottleneck on the data throughput at speeds above 30 km/h. For higher speeds the system behaves erratically by repeating the same last good frame, constantly until the speed is lowered to a sustainable data throughput again.

**Figure 8:**
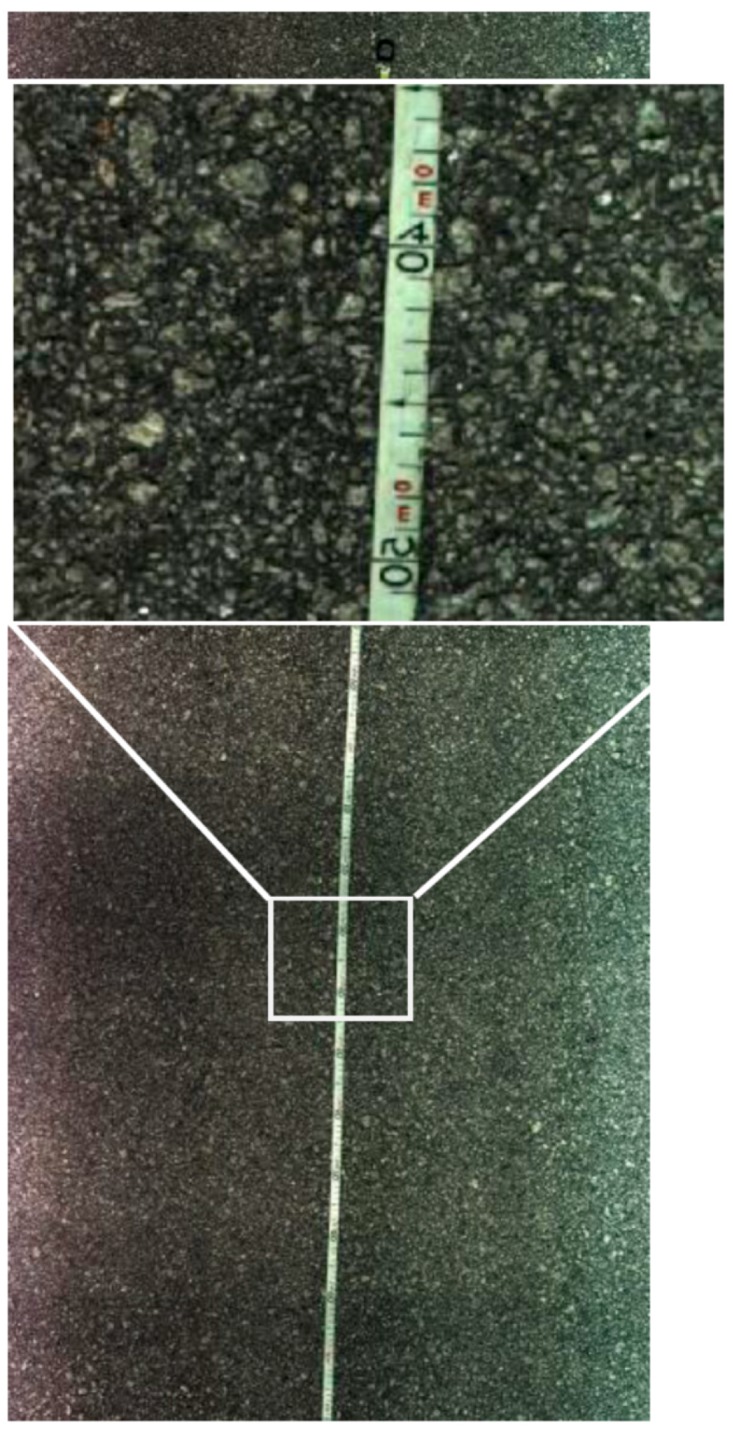
Set of stitched images with a scanned tape measure on the road and a particular zoomed area showing sub-millimeter resolution.

Figure 8 shows an extract from a sequence of images of the scanned road surface at 20 km/h, allowing the quality analysis of the image and its resolution. As it can be seen in the zoomed area of Figure 8, features smaller than one millimeter can be easily recognized. On this test, a tape measure was placed on the road and scanned at different travel speeds. The zoomed area was chosen in between two consecutive frames and a good stitching is made without any visible gaps or repetitions.

### 3.2. Scanning over Amphibian Models

**Figure 9:**
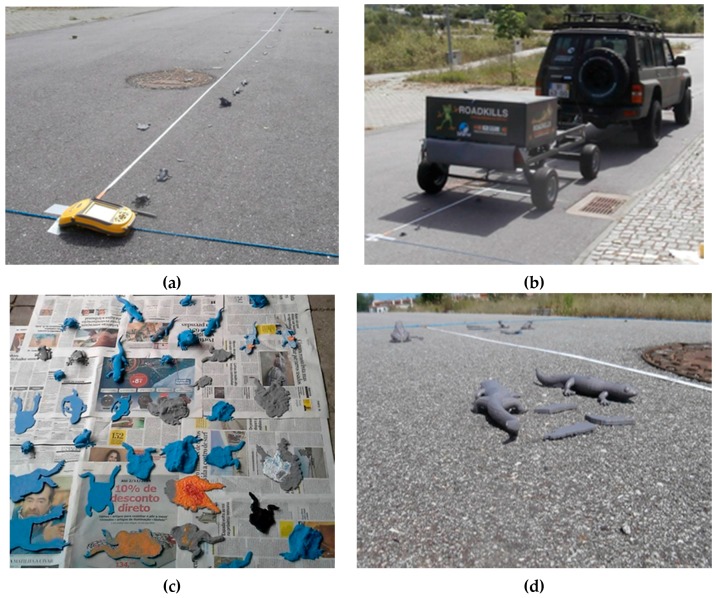
Images from the 3D models scanning tests: (**a**) Setup on asphalt with tape measure and precision GPS positioning of the specimens; (**b**) Scanning in progress; (**c**) Developed 50 specimens in 3D printed plastic; (**d**) Perspective view of the specimens on the road and their respective differences in height.

Tests were also performed by scanning 50 tridimensional (3D) specimens of printed models of amphibian casualties. They were positioned on 20 m of road as shown in Figure 9. The tests consisted on 10 scans over this setup at a constant speed of 20 km/h, in three different arrangements of the specimens. On the first trial the specimens were georeferenced with an accuracy of 1 cm. On the second trial they were grouped together on different groups. On the third trial they were positioned randomly over the cobblestone road.

## 4. Discussion

After performing various tests, the results have shown some points that needed to be addressed. An important phenomenon was found and it is visible in Figure 8. Some slight gradient changes in the pixel light intensity are clearly visible in the images. Darker areas followed by lighter areas like cast shadows are visible throughout the sequences. It is important to observe at this point since it is easier to notice this feature on a long image sequence as in Figure 8.

**Figure 10:**
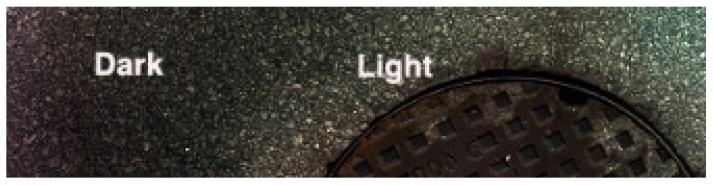
Darker and lighter areas of a scanned image and also found in many other images.

**Figure 11:**
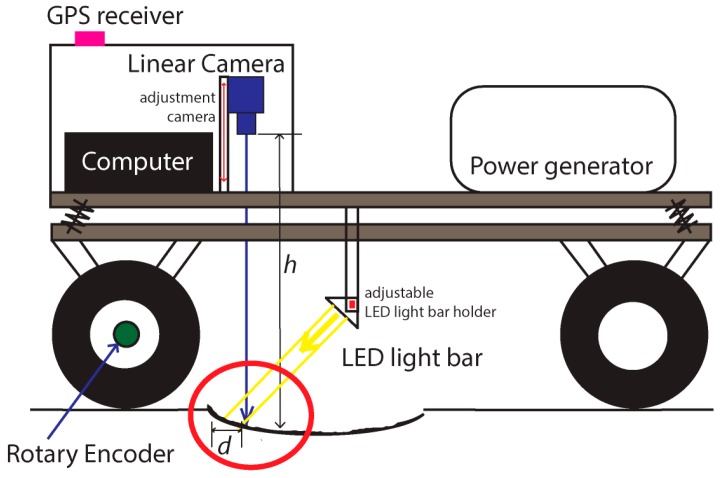
Road irregularities producing misalignments between the light line and the camera scanned line.

The same feature can be observed in Figure 10 with a darker area on the left side and a lighter area on the right side of the image. This phenomenon was related to irregularities in the road surface. Any bump or pothole in the road will interfere with the perfect alignment of the light and the calibrated point for the line scanning. Figure 11 shows a diagram demonstrating this possible cause. Two problems can be observed: (a) the distance h of the camera to the ground increases during the road depression making the acquired pixels in that area subject to be in a different focal plane; (b) the lighting that should be centered in the focal point is shifted originating therefore light intensity variations.

**Figure 12:**
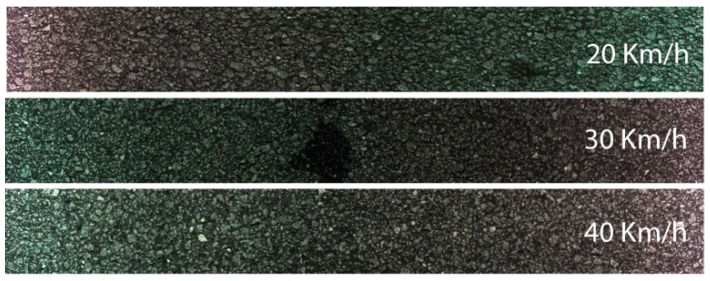
Image acquired at various travel speeds showing no loss or deterioration of the image quality.

Another important result is related with the data throughput bottleneck as mentioned earlier. Over speeding results in a disruption of the continuous sequence of image acquisition. At speeds above 30 km/h some errors were detected, however the results were considered valid for the frames considered correct and in sequence. Figure 12 shows the results of three tests performed at speeds of 20, 30 and 40 km/h. Although at the highest speed the system could not cope with a continuous recording, the non-repetitive captured images are valid and still show good quality.

The system was developed with two separated parts (top and bottom), with shock absorbers connecting both parts to reduce vibration of the top part to the minimum. All the sensitive parts are located on the top part such as the camera, light and computer. By the image quality of the acquired frames at different speeds and in heavily damaged roads, it is clear that the mechanical suspension is taking its role properly.

The 1 m image width was found to be enough for the purpose of detection of amphibian road-kills. What it was defined by the project team was that the driving trajectory must follow as much as possible the car wheel tracks centered with the scanning system. Road-killed amphibians are typically found in these tracks because it is where the car wheels normally pass on. Multiple passes can be done on the same route at different road positions, in order to acquire the whole extension of the road width, if necessary. Another possibility is by replicating the acquisition system and illumination to the desired width. If the resolution can be compromised, then the same camera can be utilized by changing the camera lens or elevating the camera position to capture a wider image. Nevertheless, the illumination system must be increased proportionally.

Above all, when compared to other systems, the developed scanner uses similar operation principles but with different technologies. High resolution color images are obtained at speeds of 30 km/h with 250 μm/pixel resolution, with similar results during day and night, in rain or foggy weather. With lower scanning resolutions, the system can be used with higher scanning speeds (60 km/h for 500 μm/pixel and 120 km/h for 1 mm/pixel). As explained earlier, laser technology is the basis of existing scanning systems and it is not applicable for this project for the already presented reasons.

In the case of the use of a line scan camera, line grouping is achieved by the hardware and consecutive images are perfectly matched and aligned. When compared to frame cameras, camera lens barrel distortion is only produced on the width axis whereas on frame cameras the distortion is produced in both axes. By stitching the consecutive images, it is noticeable where the images are joined. Thus, image post-processing is necessary to correct this distortion before stitching images. This is not necessary with the line scan camera system. Camera synchronization for consecutive frame capturing also posed some issues when frame cameras were tested. With a line scan camera this problem does not occur.

### Computer Vision Algorithms

**Figure 13:**
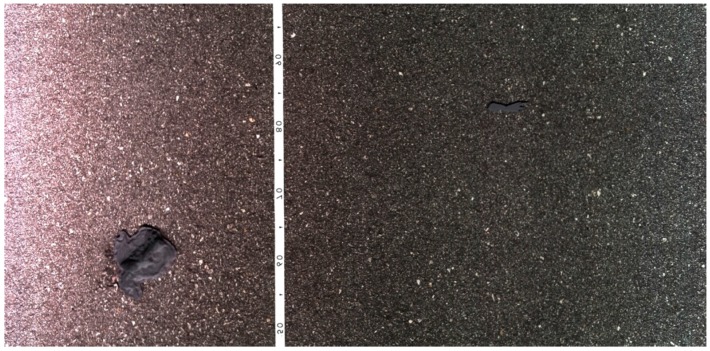
Scanned 3D model of amphibian casualties on asphalt, body part on the left and leg on the right.

**Figure 14:**
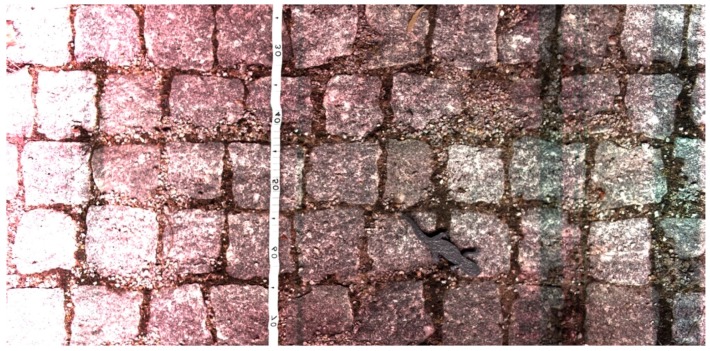
Scanned 3D model of an amphibian on a cobblestone road.

From the 3D amphibian model tests, the data was analyzed and it was supplied to train the detection algorithms with specimens used as positive search material to the database. The results using the Haar classifier method of computer vision were promising, showing more than 65% of successful findings when the files were post-processed by the computer vision algorithms. Figure 13 shows a sampled image from this trial on the asphalt and Figure 14 from the cobblestone road. On the asphalt, the left specimen is clearly seen by the human eye but on the right side of the same image the small leg model is very difficult to differentiate from the floor color. The algorithm was not able to detect it and thus it still needs to be improved, possibly by more training with better images. On the cobblestone the specimen was also not detected but many false positives were generated. Cobblestone roads are a challenge for computer vision algorithms due to their natural source of shapes in between the stones, resembling smashed animals.

**Figure 15:**
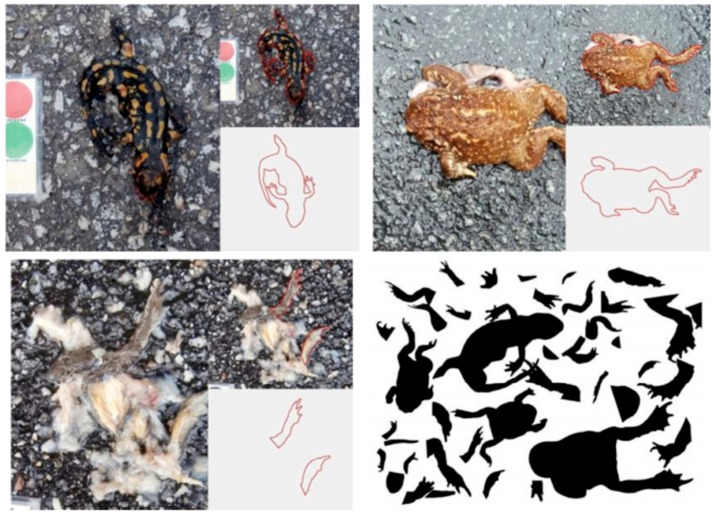
Pictures of amphibian casualties and the extracted contours of the visible detectable parts.

The algorithms are being trained with a database that was built based on casualty pictures as positive search material. Also, from each casualty, the animal contours were extracted and added to the database as shown in Figure 15. When in a casualty picture only the limbs are detectable, this contour is isolated and added to the database. This provides a more robust detection since in many situations, only the small hind limbs are visible from a smashed amphibian as it is visible in Figure 15.

**Figure 16:**
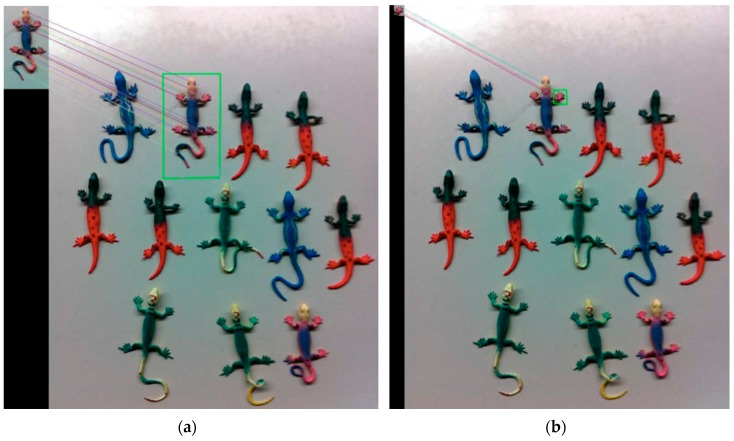
Template search using the SIFT algorithm: (**a**) Full body template of a specimen; (**b**) Right front claw of the specimen.

Other computer vision approaches are being developed by the use of known feature detection algorithms such as the Scale Invariant Feature Transform (SIFT) [22] and its successors. Figure 16 shows some tests performed on laboratory samples and the obtained results. This figure shows two instances of the searched part of one of the specimens used. In Figure 16a a complete specimen was searched and its template is located on the top left corner of the image. It was detected in the main image where other specimens were also present. In Figure 16b, the same search was performed but this time to the right claw of the specimen. This template was also found in the main image. The algorithm has shown to be efficient on feature detection although in both cases, it took around three seconds for a single frame search.

The computer vision algorithms are now under development and improvement since they are the next major step forward on the application of the developed sensor. Besides the readiness of the algorithms, the system provides valuable sensing data to the biologists in their off-line analysis of a scanned road.

## 5. Conclusions

The aim of this study was to develop, build and test a vehicle capable of acquiring images of road surfaces, with an acceptable resolution to detect small details of amphibian bodies on the road. A complete road scanner system based on a car trailer was developed, and field tests were conducted. The results suggest that the main proposed objective was achieved. Upon completion of this work, there are many lessons that can be drawn for future improvement implementations in the developed prototype.

Regarding the structure of the scanner, it was found that it meets the requirements for the purpose for which it was intended. The power generator solves the energy needs, while for short runs, batteries can be used instead to produce enough power for the whole solution. The actual complete system has a power consumption of around 800 W.

In the tests conducted at different travel speeds, the results were very satisfactory at speeds of up to 30 km/h. One of the main concerns on developing this solution was the uncertainty if the lighting system would be sufficient to obtain good quality images, in daylight and at night scenarios. The conducted tests have shown that the lighting system exceeded the expectations, as the images taken in different scenarios are almost indistinguishable. In other words, if the lighting is well framed and set with the camera line, then it is powerful enough to override sunlight and it is immune to day and night scene changes.

The trials with the 3D models of amphibians, along with the algorithm training and detection, have shown promising results but much more is still necessary to reduce the false positives in all types of roads. Several kilometers of roads were already scanned and they are available for consultation at the project’s website [23].

The presented work is registered for a patent. It is not just a theoretical approach but a fully working developed prototype with the technical details presented here. Its novelty relies in the use of a combination of technologies such as the high resolution trichromatic line scan camera, powerful LED white light for controlled illumination, precise point positioning GPS coordinates (below 1 m error), a rotary incremental encoder with 5000 points per revolution to pace the acquisition, an autonomous energy supply, computer and storage space, all inside a specially designed trailer with shock absorption to provide good quality images. The system works outdoors in all weathers as proven by the realized tests.
